# Clinical risk score for central precocious puberty among girls with precocious pubertal development: a cross sectional study

**DOI:** 10.1186/s12902-021-00740-7

**Published:** 2021-04-20

**Authors:** Jingyu You, Xianying Cheng, Xiaojing Li, Mingqing Li, Li Yao, Feihong Luo, Ruoqian Cheng, Li Xi, Jiangfeng Ye

**Affiliations:** 1 Department of Pediatric Endocrinology and Inborn Metabolic Diseases, Children’s Hospital, Fudan University, 399 Wanyuan Road, Shanghai, 201102 China; 2 Department of Ultrasonography, Children’s Hospital, Fudan University, Shanghai, 201102 China; 3 Institute of Obstetrics and Gynecology, Obstetrics and Gynecology Hospital, Fudan University, Shanghai, 200011 China; 4 Division of Obstetrics and Gynaecology, KK Women’s and Children’s Hospital, Singapore, 229899 Singapore

**Keywords:** Central precocious puberty, GnRH stimulation test, Risk score model

## Abstract

**Background:**

The gold standard for the diagnosis of central precocious puberty (CPP) is gonadotropin-releasing hormone (GnRH) or GnRH analogs (GnRHa) stimulation test. But the stimulation test is time-consuming and costly. Our objective was to develop a risk score model readily adoptable by clinicians and patients.

**Methods:**

A cross-sectional study based on the electronic medical record system was conducted in the Children’s Hospital, Fudan University, Shanghai, China from January 2010 to August 2016. Patients with precocious puberty were randomly split into the training (*n* = 314) and validation (*n* = 313) sample. In the training sample, variables associated with CPP (*P* < 0.2) in univariate analyses were introduced in a multivariable logistic regression model. Prediction model was selected using a forward stepwise analysis. A risk score model was built with the scaled coefficients of the model and tested in the validation sample.

**Results:**

CPP was diagnosed in 54.8% (172/314) and 55.0% (172/313) of patients in the training and validation sample, respectively. The CPP risk score model included age at the onset of puberty, basal luteinizing hormone (LH) concentration, largest ovarian volume, and uterine volume. The C-index was 0.85 (95% CI: 0.81–0.89) and 0.86 (95% CI: 0.82–0.90) in the training and the validation sample, respectively. Two cut-off points were selected to delimitate a low- (< 10 points), median- (10–19 points), and high-risk (≥ 20 points) group.

**Conclusions:**

A risk score model for the risk of CPP had a moderate predictive performance, which offers the advantage of helping evaluate the requirement for further diagnostic tests (GnRH or GnRHa stimulation test).

**Supplementary Information:**

The online version contains supplementary material available at 10.1186/s12902-021-00740-7.

## Background

Precocious puberty, defined as the onset of pubertal development before age 8 years in girls and 9 years in boys [1], has a prevalence of 0.43% in China and 0.01–0.02% in America girls [2, 3]. The early onset of puberty may impair children’s normal physical and psychosocial development [4–6]. However, only cases of central precocious puberty (CPP) may need a gonadotropin-releasing hormone analogs (GnRHa) therapy [1]. Although peripheral precocious puberty (PPP) will lead to central precocious puberty without optimal treatment, some pubertal development with no activation of the hypothalamic-pituitary-gonadal axis (HPGA) may regress or stop progressing without treatment, which accounted for about 50% of cases of precocious puberty [1]. In addition, with increased awareness of the importance of early treatment of CPP, more and more females with subtle signs of precocious puberty were diagnosed as precocious pubertal development [7]. Therefore, to distinguish CPP from PPP and benign variants of sexual precocity is of great importance.

The gold standard for the diagnosis of CPP is gonadotropin-releasing hormone (GnRH) or GnRHa stimulation test [1, 7]. But the stimulation test is time-consuming and costly [8]. To avoid the testing of the stimulated luteinizing hormone (LH) and follicle-stimulating hormone (FSH) concentration, baseline LH has been suggested to be used for diagnosis [9]. However, its generalization is limited by variability among studies and the small sample size of previous studies [8–13]. Pelvic ultrasonography as a part of the initial diagnostic evaluation of CPP is convenient [14–16]. But ovarian and uterine volume has a substantial overlap among girls in prepubertal and pubertal stages [7]. In addition, ovaries and uterus volume enlargement are end-organ effects caused by the gonadotropin stimulation, which suggested that pelvic ultrasonography was a highly specific but less sensitive indicator for CPP [16].

The objective of this study was to develop and validate a risk score model to predict the risk of CPP based on readily available clinical features and pelvic ultrasonography. The risk score could help make decisions on the need for further GnRH (GnRHa) stimulation test.

## Methods

### Study population

We performed a cross-sectional study based on the electronic medical record system (EMRS) in the Children’s Hospital, Fudan University, Shanghai, China. The EMRS systematically collected information on patient’s demographics, medical history, results of physical examination and laboratory test, radiology images, diagnosis, and treatment each time they visited the hospital. The sample was selected from the database including all patients who came to the hospital from January 2010 to August 2016. Patients were included according to the criteria as follows: (1) girls with a diagnosis of precocious puberty; (2) girls at the age of 8 years old or less when she was diagnosed; (3) hormone assay (including GnRHa stimulation test) and pelvic ultrasonography performed in the Children’s Hospital, Fudan University; (4) pelvic ultrasonography performed within 1 week of the GnRHa stimulation test (before the GnRHa stimulation test). Patients with secondary precocious puberty (precocious pubertal manifestations are the secondary changes of primary lesions), e.g. precocious puberty due to CNS lesions (congenital or acquired) or ovarian cyst were not included in the study, because HPGA, target organs and the results of GnRHa stimulation test may all be affected by the primary diseases. In addition, the treatment of the secondary precocious puberty is quite different from the idiopathic CPP, which will not mainly base on the results of GnRHa stimulation test.

This study was approved by the Ethics Committees of the Children’s Hospital, Fudan University, Shanghai, China.

### Gold standard

GnRHa stimulation test was the gold standard for the diagnosis of CPP [1, 7]. Patients with stimulated peak LH ≥ 5 IU/L, and peak LH-to-FSH ratio ≥ 0.6 were diagnosed as CPP [1, 8–10, 17]. Details of the GnRHa stimulation test have been published elsewhere [18]. LH and FSH concentration was measured using electrochemiluminescence assay (COBASE 602, Roche, Switzerland). The limit of detection (LOD) of LH and FSH was 0.2 IU/L. Stimulated LH, basal and stimulated FSH concentration was above the LOD in all participants. Basal LH level was below the LOD in 1.8% (11/627) patients [2.5% (8/314) and 1.0% (3/313) in the training and validation sample, respectively].

### Pelvic ultrasound evaluation

Transabdominal ultrasonography was performed utilizing a curvilinear 2–7 MHz probe. All pelvic ultrasonograms were obtained with Philips IU22 ultrasound units equipped with duplex/color-flow Doppler broad bandwidth transducers (Phillips, Netherlands). The pediatric radiologist had no information on the results of the GnRHa stimulation test. Ovarian volume for each side was calculated using the ellipse volume formula: 0.5233*length*depth*breadth. Average ovarian volume was calculated as: (right ovary volume + left ovary volume)/2. The largest and smallest ovarian volume was defined as the larger and smaller volume between the right and left ovary volume. Uterine volume was calculated according to the same ellipse volume formula. The values of sonographic characteristics were stratified into categories (ovarian volume: < 1 mL, 1- < 2 mL, and ≥ 2 mL; uterine length: < 3 cm, 3- < 4 cm, and ≥ 4 cm; uterine volume: < 3 mL, 3- < 4 mL, and ≥ 4 mL; uterine configuration with the thickness of endometrial stripe: < 0.2 cm and ≥ 0.2 cm) [16].

### Medical history, physical examination and bone age

A complete medical history and results of the physical examination were extracted from the database. Breast and pubic hair development was assessed according to the Tanner staging criteria [1]. The bone age (BA) was measured using the Greulich PyIe (GP) method [19].

### Statistical analysis

A random sample including one half of the patients was selected to develop a clinical prediction model (training sample), leaving the other half of the patients for validation (validation sample). We first compared the clinical characteristics and pelvic ultrasonography between the training and validation sample using a quantitative (*t* test or Wilcoxon rank sum test) or qualitative (*χ*^*2*^ test) test as appropriate. Then we built crude logistic regression models to evaluate the association between potential predictors and CPP. A total of 30 variables containing information on medical history, progression of pubertal manifestations, basal hormone level, and pelvic ultrasonography were selected as potential predictors according to previous studies (See Additional file 1[Additional Table 1]) [1, 7, 16]. Variables with *P* values less than 0.20 in the univariate logistic regression models entered the multivariable logistic regression model. The prediction model was selected using forward stepwise analysis (variables with *P* = 0.05 were included, while those with *P* > 0.10 were removed). Performance of the selected model was assessed using C-index, calibration based on Hosmer-Lemeshow test [20]. We performed the internal validation using bootstrap resampling [21].

A risk score model based on the final logistic regression model was derived using the method proposed by Sullivan et al. [22]. In the risk score system, the risk for CPP was demonstrated by total points which were calculated according to the logistic regression model. The statistical methods were described in detail in Additional file 2. The performance of risk score model was measured using C-index, calibration, sensitivity, specificity, positive likelihood ratio (LR+) and negative likelihood ratio (LR-) [20]. The cut off points of the risk score were selected according to the risk score distribution and adjusted with consideration for the convenience of clinical adoption. A team of two experienced pediatric endocrinologists, two pediatric radiologists, and an epidemiologist reached a consensus on the cut off points. Validation was performed in the other half of the patients. Performance of the CPP risk score model in the validation sample was measured as well [20].

Statistical analyses were performed using SAS statistical software version 9.2 (SAS Institute Inc., Cary, NC, USA).

## Results

### Patient characteristics

**Fig. 1:**
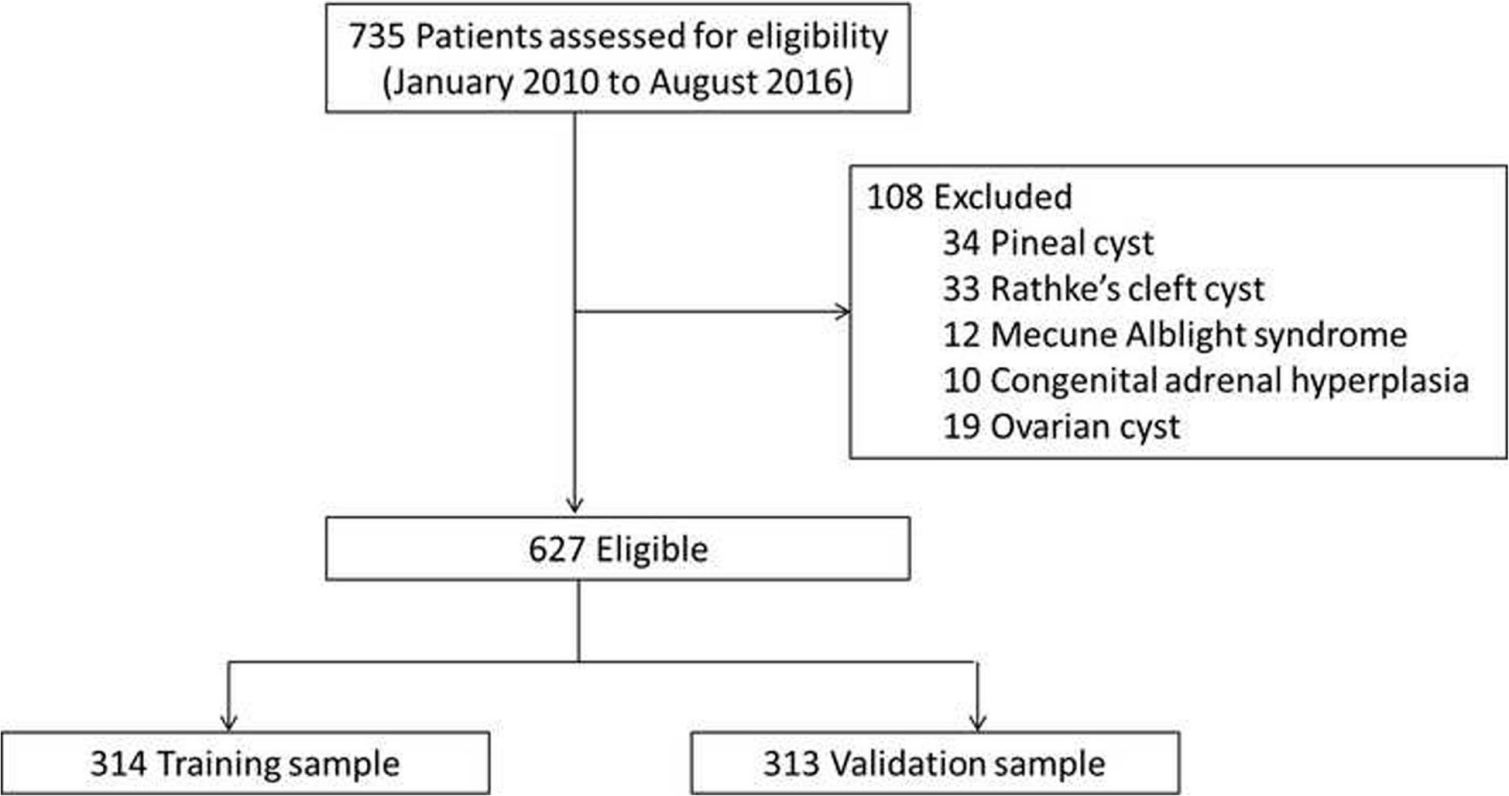
Study subjects flow chart

A total of 735 patients met the inclusion criteria. Patients with pineal cyst (*n* = 34), Rathke’s cleft cyst (*n* = 33), Mecune Alblight syndrome (*n* = 12), congenital adrenal hyperplasia (*n* = 10), and ovarian cyst (*n* = 19) were excluded. Finally, 627 patients were included and randomly separated into the training sample (*n* = 314) and validation sample (*n* = 313) (Fig. 1).

The mean age of the participants was 7.5 years [95% confidence interval (CI), 7.4–7.7 years]. The average disease duration was 1.0 years (95% CI, 1.0–1.2 years). CPP was diagnosed in 54.8% (172/314) and 55.0% (172/313) of patients in the training and validation sample, respectively. Patients did not show significant difference of clinical or pelvic ultrasonography characteristics in the training and validation sample except for the family history of CPP. Detailed description was showed in Table 1.

**Table 1:** Clinical and ultrasonography characteristics of patients with premature sexual development in training and validation samples

	Training (*n* = 314)	Validation (*n* = 313)	*P* Value
Central precocious puberty (%)	172 (54.8)	172 (55.0)	0.9649
Clinical characteristics			
Age at onset of puberty [mean (SD), year]	6.5 (1.6)	6.5 (1.6)	0.8132
Chronological age [mean (SD), year]	7.5 (1.6)	7.6 (1.7)	0.9460
Bone age [mean (SD), year]	9.6 (7.5)	8.9 (2.3)	0.1415
Bone age/ Chronological age (SD)	1.2 (0.2)	1.2 (0.2)	0.8146
Duration of disease [mean (SD), year]	1.1 (0.9)	1.0 (0.8)	0.7547
Family history of central precocious puberty (%)	5 (1.6)	0 (0.0)	0.0250
Tanner stage for breast development			
Left (%)			0.5959 ^a^
I	9 (2.9)	9 (2.9)	
II	200 (64.5)	211 (68.5)	
III	98 (31.6)	87 (28.3)	
IV	3 (1.0)	1 (0.3)	
Right (%)			0.4052 ^a^
I	12 (3.9)	8 (2.6)	
II	196 (63.2)	211 (68.5)	
III	99 (31.9)	88 (28.6)	
IV	3 (1.0)	1 (0.3)	
Tanner stage for pubic hair development (%)			0.1860 ^a^
I	277 (88.2)	282 (90.1)	
II	32 (10.2)	31 (9.9)	
III	4 (1.3)	0 (0.0)	
IV	1 (0.3)	0 (0.0)	
Height [mean (SD), cm]	130.0 (11.6)	129.9 (12.6)	0.8986
Weight [mean (SD), kg]	28.4 (6.8)	28.8 (7.3)	0.5405
BMI [mean (SD), kg/m^2^]	16.7 (2.2)	16.9 (2.4)	0.3691
LH [Median (IQR), IU/L]			
Baseline	0.43 (0.17, 1.07)	0.43 (0.18, 1.02)	0.6364
Stimulated	10.45 (5.01, 21.36)	9.55 (4.80, 24.97)	0.9503
FSH [mean (SD), IU/L]			
Baseline	3.7 (2.3)	3.6 (2.2)	0.6839
Stimulated	16.7 (8.0)	17.0 (7.8)	0.6182
LH/FSH [Median (IQR)]			
Baseline	0.14 (0.08, 0.29)	0.14 (0.07, 0.30)	0.5607
Stimulated	0.75 (0.38, 1.46)	0.72 (0.32, 1.45)	0.7394
Estradiol [Median (IQR), pg/mL]	15.0 (8.0, 29.5)	14.0 (7.0, 26.8)	0.4737
hCG [Median (IQR), IU/L]	0.08 (0.00, 0.23)	0.05 (0.00, 0.21)	0.4128
Prolactin [mean (SD), ng/mL]	10.7 (7.7)	9.7 (5.8)	0.1042
DHEAS [mean (SD), μg/dL]	53.3 (38.3)	53.5 (39.6)	0.9594
Testosterone [Median (IQR), ng/dL]	2.4 (0.0, 16.3)	0.0 (0.0, 15.1)	0.3793
Cortisol [mean (SD), μg/dL]	8.3 (4.9)	8.3 (4.7)	0.9896
ACTH [Median (IQR), pg/mL]	24.2 (17.8, 33.0)	24.5 (18.0, 34.0)	0.7214
Total triiodothyronine [mean (SD), ng/dL]	139.8 (24.3)	136.5 (25.1)	0.0982
Free triiodothyronine [mean (SD), pg/mL]	3.9 (0.6)	3.8 (0.6)	0.8073
Total thyroxine [mean (SD), μg/dL]	9.0 (1.8)	9.1 (1.8)	0.3588
Free thyroxine [mean (SD), ng/dL]	1.0 (0.2)	1.0 (0.2)	0.6164
TSH [mean (SD), μIU/mL]	2.4 (1.4)	2.2 (1.2)	0.1302
Pelvic sonogram			
Ovarian volume			
Average ovarian (%)	1.9 (0.9)	1.9 (0.9)	0.6001
< 1 mL	40 (12.7)	44 (14.1)	0.8866
1- < 2 mL	157 (50.0)	155 (49.5)	
≥ 2 mL	117 (37.3)	114 (36.4)	
Largest ovarian (%)			
< 1 mL	31 (9.9)	34 (10.9)	0.8914
1- < 2 mL	142 (45.2)	137 (43.8)	
≥ 2 mL	141 (44.9)	142 (45.4)	
Smallest ovarian (%)			
< 1 mL	67 (21.3)	73 (23.3)	0.8138
1- < 2 mL	160 (51.0)	153 (48.9)	
≥ 2 mL	87 (27.7)	87 (27.8)	
Uterine			
Length			
< 3 cm	278 (88.5)	287 (91.7)	0.1774 ^a^
3- < 4 cm	36 (11.5)	25 (8.0)	
≥ 4 cm	0 (0.0)	1 (0.3)	
Volume			
< 3 mL	244 (77.7)	251 (80.2)	0.7287
3- < 4 mL	42 (13.4)	36 (11.5)	
≥ 4 mL	28 (8.9)	26 (8.3)	
Endometrium visible (%)	40 (12.7)	40 (12.8)	0.9878

*BMI* body mass index, *LH* luteinizing hormone, *FSH* follicle-stimulating hormone, *hCG* human chorionic gonadotropin, *DHEAS* dehydroepiandrosterone sulfate, *ACTH* adrenocorticotropic hormone, *TSH* thyroid - stimulating hormone, *IQR* interquartile range

^a^Calculated using Fisher’s exact test

### Training sample

**Fig. 2:**
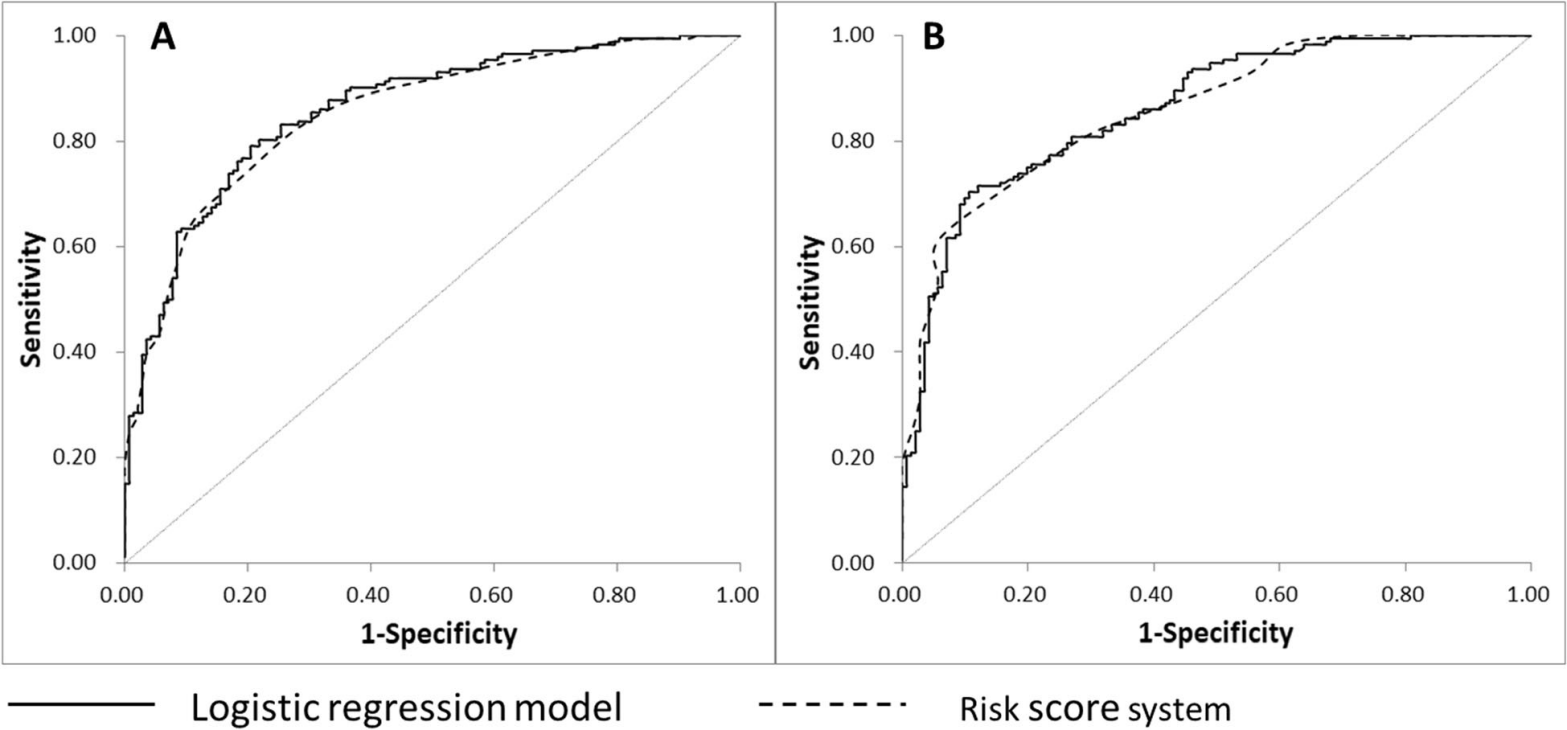
Receiver operating characteristic curves of the prediction model and risk score system. **a** Training sample. **b** Validation sample

The crude relationship between potential predictors and CPP was showed in Additional file 1 (Additional Table 1). A total of 21 variables with *P* values less than 0.20 entered the multivariable logistic regression model. After a forward stepwise selection, a final model including four predictors (age at the onset of puberty, basal LH, largest ovarian volume, and uterine volume) was selected (Additional file 1 [Additional Table 2]). The variance inflation factor was less than 2.0 for all predictors, indicating there was no linear relationship between predictors. The C-index was 0.86 [95% CI, 0.82–0.90; Fig. 2]. Hosmer-Lemeshow test demonstrated goodness of fit for the prediction model (*P* = 0.49). The calibration plot showed an intercept of − 0.01, and a slope of 1.01 (Additional file 1 [Additional Figure 1]). A bootstrap analysis (resampling the model 300 times) showed a corrected C-index of 0.86.

Points were assigned to each category of the predictors (Table 2). The risk scores with a range of 0 to 33 linearly correlated with the CPP risk estimates (*r* = 0.96, *P* < 0.0001, Table 3). The proportion of patients with CPP in each group of the risk score point was showed in Table 3. C-index for the risk score system was 0.85 (95% CI, 0.81–0.89, Fig. 2). Calibration plot showed an intercept of − 0.02, and a slope of 1.02 (Additional file 1 [Additional Figure 1]).

**Table 2:** Determine points associated with each of the categories of each risk factor

Predictors	Categories	Reference value (***W***_***ij***_)	β_**i**_	β_**i**_ (***W***_***ij***_-***W***_***iREF***_)	Points_**ij**_ = β_**i**_ (***W***_***ij***_-***W***_***iREF***_)/B ^***a***^
Age at onset of puberty (years)	< 1	0.5 = *W*_*1REF*_	0.45	0	0
	1 - < 2	1.5		0.45	1
	2 - < 3	2.5		0.90	3
	3 - < 4	3.5		1.35	4
	4 - < 5	4.5		1.80	6
	5 - < 6	5.5		2.25	7
	6 - < 7	6.5		2.70	8
	7 - < 8	7.5		3.15	10
Basal LH (IU/L)	< 0.2	0.1 = *W*_*2REF*_	1.63	0	0
	0.2 - < 0.4	0.3		0.326	1
	0.4 - < 0.6	0.5		0.652	2
	0.6 - < 0.8	0.7		0.978	3
	0.8 - < 1.0	0.9		1.304	4
	1.0 - < 1.2	1.1		1.630	5
	1.2 - < 1.4	1.3		1.956	6
	1.4 - < 1.6	1.5		2.282	7
	1.6 - < 1.8	1.7		2.608	8
	1.8 - < 2.0	1.9		2.934	9
	2.0 - < 2.2	2.1		3.260	10
	2.2 - < 2.4	2.3		3.586	11
	2.4 - < 2.6	2.5		3.912	12
	≥ 2.6	3.0		4.727	14
Largest ovarian volume	< 1 mL	1 = *W*_*3REF*_	0.66	0	0
	1- < 2 mL	2		0.66	2
	≥ 2 mL	3		1.32	4
Uterine volume	< 3 mL	1 = *W*_*4REF*_	0.85	0	0
	3- < 4 mL	2		0.85	3
	≥ 4 mL	3		1.70	5

Points associated with each category of each risk factor are computed by: Points_ij_ = β_i_ (*W*_*ij*_-*W*_*iREF*_)/B and rounded to the nearest integer

^*a*^We define the constant B for the points system (the number of regression units that will correspond to one point) as the increase in risk of CPP associated with a 0.2 (IU/L) increase in basal LH: B = 0.2*1.63 = 0.326

**Table 3:** Points system and associated risks for central precocious puberty in the training and validation sample

CPP risk category	Points total	Estimate of risk (95% CI) ^*a*^	No. with CPP/ Total No. of patients in training sample (%)	No. with CPP/ Total No. of patients in validation sample (%)
Low risk	0	0.01 (0.01, 0.03)	0/0 (−)	0/1 (0.0)
	1	0.01 (0.01, 0.04)	0/1 (0.0)	0/3 (0.0)
	2	0.02 (0.01, 0.05)	0/3 (0.0)	0/0 (−)
	3	0.03 (0.01, 0.07)	0/4 (0.0)	0/6 (0.0)
	4	0.04 (0.02, 0.08)	0/2 (0.0)	0/5 (0.0)
	5	0.05 (0.02, 0.10)	1/3 (33.3)	0/1 (0.0)
	6	0.07 (0.04, 0.13)	0/6 (0.0)	0/3 (0.0)
	7	0.09 (0.05, 0.16)	0/3 (0.0)	0/3 (0.0)
	8	0.12 (0.08, 0.20)	1/9 (12.5)	0/6 (0.0)
	9	0.16 (0.11, 0.24)	2/9 (22.2)	0/11 (0.0)
Medium risk	10	0.21 (0.15, 0.29)	4/19 (21.1)	3/19 (15.8)
	11	0.27 (0.21, 0.35)	5/21 (23.8)	10/19 (52.6)
	12	0.34 (0.27, 0.42)	7/28 (25.0)	13/37 (35.1)
	13	0.42 (0.35, 0.49)	10/24 (41.7)	7/19 (36.8)
	14	0.50 (0.43, 0.57)	16/29 (55.2)	17/35 (48.6)
	15	0.58 (0.51, 0.65)	14/25 (56.0)	17/32 (53.1)
	16	0.66 (0.58, 0.72)	15/19 (79.0)	14/14 (100.0)
	17	0.72 (0.65, 0.79)	9/11 (81.8)	14/17 (82.4)
	18	0.78 (0.70, 0.85)	13/15 (86.7)	5/6 (83.3)
	19	0.83 (0.76, 0.89)	7/10 (70.0)	12/12 (100.0)
High risk	20	0.87 (0.80, 0.92)	9/10 (90.0)	6/6 (100.0)
	21	0.91 (0.84, 0.95)	8/9 (88.9)	7/8 (87.5)
	22	0.93 (0.87, 0.96)	3/3 (100.0)	5/6 (83.3)
	23	0.95 (0.90, 0.98)	6/8 (75.0)	4/5 (80.0)
	24	0.96 (0.92, 0.98)	10/11 (90.9)	6/7 (85.7)
	25	0.97 (0.93, 0.99)	5/5 (100.0)	3/3 (100.0)
	26	0.98 (0.95, 0.99)	8/8 (100.0)	5/5 (100.0)
	27	0.99 (0.96, 0.99)	2/2 (100.0)	4/4 (100.0)
	28	0.99 (0.97, 1.00)	4/4 (100.0)	3/3 (100.0)
	29	0.99 (0.97, 1.00)	3/3 (100.0)	1/1 (100.0)
	30	0.99 (0.98, 1.00)	2/2 (100.0)	1/1 (100.0)
	31	1.00 (0.98, 1.00)	6/6 (100.0)	7/7 (100.0)
	32	1.00 (0.99, 1.00)	0/0 (−)	0/0 (−)
	33	1.00 (0.99, 1.00)	2/2 (100.0)	8/8 (100.0)

*a* $$ \hat{p}=\frac{1}{1+\exp \left(-{\sum}_{i=0}^p{\beta}_i{X}_i\right)} $$, $$ {\sum}_{i=0}^p{\beta}_i{X}_i $$ = − 6.42 + 0.45*0.5 + 1.63*0.1 + 0.66*1 + 0.85*1 + B*(Point total)

We define the constant B for the points system (the number of regression units that will correspond to one point) as the increase in risk of CPP associated with a 0.2 (IU/L) increase in basal LH: B = 0.2*1.63 = 0.326

The risk scores were further divided into three tertiles. Patients were categorized into the low, middle, and high risk population accordingly (low risk: < 10 points; medium risk: 10–19 points; high risk: ≥ 20 points) (Table 3). Proportion of CPP patient in the low-, medium-, and high-risk population was 10% (4/40), 49.8% (100/201), and 93.2% (68/73), respectively. In the low-risk population, the sensitivity was 97.8% (95% CI, 95.3–99.5%), the specificity was 24.8% (95% CI, 18.2–32.6%), the LR- was 0.09 (95% CI, 0.02–0.20), and the negative predictive value was 90.2% (95% CI, 79.2–97.7%). In the high-risk population, the specificity was 96.6% (95% CI, 92.9–99.2%), the sensitivity was 39.6% (95% CI, 32.0–46.2%), the LR+ was 12.0 (95% CI, 5.49–48.9), and the positive predictive value was 93.3% (95% CI, 86.5–98.4%; Table 4).

**Table 4:** Predictive ability of the risk score system for CPP in training and validation samples

	Training Sample (*n* = 314)	Validation Sample (*n* = 313)
C-index (95%CI)	0.85 (0.81, 0.89)	0.86 (0.82, 0.90)
Calibration	a = −0.02, b = 1.02	a = −0.02, b = 1.06
Cutoff point = 10		
Sensitivity (%, 95CI)	97.8 (95.3, 99.5)	100.0 (−)
Specificity (%, 95CI)	24.8 (18.2, 32.6)	27.7 (20.2, 34.9)
Positive likelihood ratio (95%CI)	1.30 (1.20, 1.46)	1.38 (1.25, 1.54)
Negative likelihood ratio (95%CI)	0.09 (0.02, 0.20)	0.0 (−)
Positive predictive value (%, 95CI)	61.2 (55.9, 67.0)	62.6 (56.8, 68.6)
Negative predictive value (%, 95CI)	90.2 (79.2, 97.7)	100.0 (−)
Cutoff point = 20		
Sensitivity (%, 95CI)	39.6 (32.0, 46.2)	34.8 (27.8, 42.1)
Specificity (%, 95CI)	96.6 (92.9, 99.2)	97.3 (94.2, 100.0)
Positive likelihood ratio (95%CI)	12.0 (5.49, 48.9)	12.6 (5.44, 48.8)
Negative likelihood ratio (95%CI)	0.63 (0.55, 0.71)	0.67 (0.59, 0.75)
Positive predictive value (%, 95CI)	93.3 (86.5, 98.4)	93.9 (86.6, 100.0)
Negative predictive value (%, 95CI)	56.9 (50.8, 62.9)	55.2 (48.8, 61.4)

### Validation sample

There were 313 patients in the validation sample. C-index was 0.86 (95% CI, 0.82–0.90%) for both logistic regression model and risk score model (Fig. 2). Calibration plot of the observed frequency of CPP patients against the predicted probability of CPP showed an intercept of − 0.02, and a slope of 1.06, suggesting acceptable calibration (Additional file 1 [Additional Figure 1]).The total risk score in the validation sample ranged from 0 to 33. The proportion of CPP patients in the low-, medium-, and high-risk population was 0.0% (0/39), 53.3% (112/210), and 93.8% (60/64), respectively (Table 4).

### Model comparison

We compared the predictive performance of models with individual predictor (age at the onset of puberty, basal LH, ovarian volume, and uterine volume) and the model with all selected predictors (Additional file 1 [Additional Table 3 and Additional Figure 2]). All the predictors are statistically significant in both training sample and validation sample. Basal LH is the most important predictor (area under the ROC curve [AUC] = 0.82 and 0.84 in the training and validation sample, respectively). The predictive performance improved further after including “age at the onset of puberty” and ovarian volume, uterine volume in the model.

## Discussion

GnRH (GnRHa) stimulation test is the gold standard for CPP. But it is time-consuming and costly [1, 7]. In this study, we developed a risk score system (4 items with a 33 - point total scale) containing information on age at the onset of puberty, basal LH concentration, and pelvic sonography for the prediction of CPP. The risk score model performed well in both training and validation sample (C-index of 0.85 and 0.86, respectively). We suggested cut off points of 10 and 20 based on the tertiles of risk scores and for the convenience of clinical adoption. The method was also used in other study [23]. The prediction model had a sensitivity of 97.8% and a LR- of 0.09 in the low risk population; it had a specificity of 96.6% and a LR+ of 12.0 in the high risk population. The stratification of the risk scores would help make the decision for the need of further diagnostic tests.

All variables in the prediction model have been demonstrated to be associated with CPP in previous studies [1, 7]. Thelarche is the first sign of puberty [24]. Premature thelarche occurred before the age of 2 years old may possibly regress completely, while premature thelarche usually leads to early puberty when it occurs after age 2 years old [25]. LH concentration is the most valuable parameter for the diagnosis of CPP. Various cut-off points of basal LH ranging from 0.1 to 1.5 IU/L had been used to evaluate the activation of HPAG, which resulted in a sensitivity and specificity ranging from 60 to 100% [8–13, 26]. The wide variations had hampered the definition of cut-off point of basal LH to discriminate CPP. In addition, basal LH was elevated after the stimulated LH, which suggested that basal LH was an indicator with a high specificity but low sensitivity [1, 9, 16]. Our findings agreed with previous studies and confirmed that the high risk score resulted from an elevated basal LH concentration and was associated with enlarged ovarian volume.

Ovaries and uterus enlargement is the end-organ effect of gonadotropin stimulation, which occurs in the late stage of puberty development (ovary development in stage 3 and uterine development in stage 4) [15, 16]. It was reported that a female with an average ovarian volume less than 2 mL has 75% chance of being prepuberty [16]. A uterine volume of greater than 2 mL has also been considered as an indicator for the diagnosis of CPP [27]. However, there was substantial overlap in ovarian and uterine volumes between girls in the prepubertal and pubertal stage, which suggested that pelvic ultrasonography alone could not be a sensitive indicator for CPP [16]. We found that largest ovarian volume is the most sensitive pelvic ultrasonography indicator. But even the largest ovarian volume could not serve as an indicator independently to discriminate CPP from PPP.

All the predictors (age at the onset of puberty, basal LH, ovarian volume, and uterine volume) were statistically significant in both univariate and multivariate predictive models. Basal LH is the most important predictor. The predictive performance improved further after including “age at the onset of puberty”, ovarian volume, and uterine volume in the model. Furthermore, inquiry about “age at the onset of puberty” and pelvic ultrasound evaluation is a part of the routine diagnostic method of CPP. The information is obtainable without extra burden on patients. A predictive model including medical history (age at the onset of puberty), basal LH, and the pelvic ultrasound evaluation is suggested to evaluate the necessity of GnRHa stimulation test.

Our study developed a risk score model for CPP including information on both basal LH and pelvic ultrasonography. Based on the stratification of the CPP risk score, we suggest that patients in the high-risk category (≥ 20 points) could be diagnosed as CPP without GnRHa stimulation test; patients with a median-risk (10–19 points) need a stimulation test for further diagnosis; patients with a low-risk CPP score (< 10 points) need to be followed for the pubertal development.

Strengths of this study included the objective assessment of pelvic ultrasonography. Pelvic ultrasonography was performed within 1 week of the GnRHa stimulation test. Radiologists had no information on the result of the diagnosis test. Moreover, a large external validation sample confirmed good predictive performance of the risk score model. To our knowledge, it is the first study that developed and validated a risk score model for the diagnosis of CPP using a large sample.

However, there are several limitations to this study. First, all patients (both training and validation sample) came from the Children’s Hospital, Fudan Univeristy. Performance of the risk score may vary in different populations, which resulted in the limitation of the generalizability. But as a collaborator of the Children’s National Medical Centre, many patients come from other cities or provinces. Given the prevalence of precocious puberty was 0.43% in China [2], the current study population with a large sample size can be considered as a representative sample of patients. Future study would benefit from the assessment of the risk score model in other clinical settings. Second, information on the puberty development was not available in the EMRS, because many patients with negative stimulation test were followed up and treated (if necessary) in other facilities near to their home. In addition, patients with positive GnRHa stimulation tests started on the GnRHa treatment immediately after being diagnosis as CPP. In the current study, we evaluated the performance of the risk score model based on the results of GnRHa stimulation test without further validation against the progressive puberty. Third, most subjects in the current study were patients with recent onset of puberty. It may not represent the complicated spectrum of precocious puberty. But patients with longer duration of pubertal development may have more pubertal manifestations than the newly onset patients. The inclusion of patients with longer disease duration may not decrease the diagnostic value of the risk score model. Fourth, LH and FSH concentration was measured using electrochemiluminescence assay with a LOD of 0.2 IU/L in this study. The LH concentration records were extracted from the medical history of the database. Variations among batches could not be avoided. Assay characteristics and interassay variations may result in a reduction of the predictive performance [1]. Fifth, the variation in the pelvic ultrasonography measurement among radiologists may also introduce bias. However, all radiologists had no information on the results of the stimulation test. The misclassification was not differential, which may result in an underestimation of the performance of the risk score model. Finally, both basal LH and pelvic ultrasonography are indicators of the activation of HPGA in the late stage, which leads to higher specificity but less sensitivity of the prediction model. Patients with high-risk score would be a major beneficiary of the risk score model. Based on the risk scores, high-risk patients could be diagnosed without GnRHastimulation test; patients at medium-risk of CPP need diagnostic test promptly; patients at the low-risk category need to be followed up.

## Conclusions

A risk score model for the risk of CPP including information on medical history, basal LH, and pelvic ultrasonography had a moderate predictive performance. The risk score model offers the advantage of helping evaluate the requirement for further diagnostic test (GnRH or GnRHa stimulation test). Validations in other clinical settings are needed before the adoption in clinical practice.

## Supplementary Information

**Additional file 1: Additional Table 1.** Association of clinical characteristics and pelvic sonographic variables with central precocious puberty in the training sample (*n* = 314). **Additional Table 2.** Selected prediction model for central precocious puberty in the training sample (*n* = 314). **Additional Figure 1.** Calibration plot for the selected logistic regression model and risk score model in the training and validation sample (A. Training sample B. Validation sample).

**Additional file 2.** Method: Risk score system.

## Data Availability

The dataset analyzed during the current study are available from the corresponding author on reasonable request. No administrative permissions were required to access the raw data.

## Abbreviations

BMI: Body mass index
CPP: Central precocious puberty
GnRH: Gonadotropin-releasing hormone
GnRHa: Gonadotropin-releasing hormone analogs
HPGA: Hypothalamic-pituitary-gonadal axis
PPP: Peripheral precocious puberty
LH: Luteinizing hormone
FSH: Follicle-stimulating hormone
EMRS: Electronic medical record system
LOD: Limit of detection
BA: Bone age
GP: Greulich PyIe
LR +: Positive likelihood ratio
LR-: Negative likelihood ratio
CI: Confidence interval
hCG: Human chorionic gonadotropin
DHEAS: Dehydroepiandrosterone sulfate
ACTH: Adrenocorticotropic hormone
TSH: Thyroid - stimulating hormone
IQR: Interquartile range
