# Supplementary material for: Clinical risk score for central precocious puberty among girls with precocious pubertal development: a cross sectional study
Source: BMC Endocr Disord. 2021 Apr 20;21:75. doi: 10.1186/s12902-021-00740-7 (PMC8056580; doi:10.1186/s12902-021-00740-7)
Supplement: Supplementary file 1 — Additional file 1: Additional Table 1. Association of clinical characteristics and pelvic sonographic variables with central precocious puberty in the training sample (n = 314). Additional Table 2. Selected prediction model for central precocious puberty in the training sample (n = 314). Additional Figure 1. Calibration plot for the selected logistic regression model and risk score model in the training and validation sample (A. Training sample B. Validation sample). [file 12902_2021_740_MOESM1_ESM.docx]

Additional Table 1 Association of clinical characteristics and pelvic sonographic variables with central precocious puberty in the training sample (n=314)

| **Predictors** | **No. of exposed in the patients with CPP (%)** | **CPP (n=172)** | **Non-CPP (n=142)** | **Odds ratio (95% CI)** | ***P* value** |
| --- | --- | --- | --- | --- | --- |
| Clinical characteristics |  |  |  |  |  |
| Age at onset of puberty [mean (SD), year] *^a^* | - | 7.0 (1.1) | 5.9 (1.8) | 1.72 (1.42, 2.08) | <0.0001 |
| Bone age [mean (SD), year] | - | 9.8 (1.4) | 8.0 (2.3) | 1.01 (0.98, 1.04) | 0.6578 |
| Bone age/ chronological age [mean (SD)] | - | 1.2 (0.2) | 1.1 (0.2) | 0.93 (0.76, 1.13) | 0.4397 |
| Family history of CPP (%) | 3 (1.7) | - | - | 1.24 (0.21, 7.54) | 0.8134 |
| Height [mean (SD), cm] ^a^ | - | 133.9 (7.8) | 125.2 (13.5) | 1.09 (1.06, 1.12) | <0.0001 |
| Weight [mean (SD), kg] *^a^* | - | 30.6 (5.6) | 25.7 (7.3) | 1.13 (1.08, 1.18) | <0.0001 |
| BMI [mean (SD), kg/m^2^] *^a^* | - | 17.0 (2.0) | 16.3 (2.4) | 1.16 (1.04, 1.30) | 0.0081 |
| Tanner stage for breast development *^a^* |  |  |  |  |  |
| Left [median (IQR)] | - | 2 (2, 3) | 2 (2, 3) | 2.34 (1.49, 3.67) | 0.0002 |
| Right [median (IQR)] | - | 2 (2, 3) | 2 (2, 3) | 2.35 (1.52, 3.64) | 0.0001 |
| Tanner stage for pubic hair development *^a^* | - | 1 (1,1) | 1 (1,1) | 2.44 (1.22, 4.88) | 0.0117 |
| Basal LH [median (IQR), IU/L] *^a^* | - | 0.88 (0.41, 1.71) | 0.20 (0.12, 0.41) | 6.61 (3.69, 11.85) | <0.0001 |
| Basal FSH [median (IQR), IU/L] *^a^* | - | 4.14 (2.53, 5.73) | 2.46 (1.55, 3.90) | 1.46 (1.28, 1.66) | <0.0001 |
| Basal LH/FSH ratio [median (IQR)] | - | 0.21 (0.13, 0.37) | 0.09 (0.05, 0.15) | 0.95 (0.82, 1.09) | 0.4467 |
| Basal estradiol [median (IQR), pg/mL] *^a^* | - | 19.0 (9.0, 36.0) | 12.0 (6.0, 21.0) | 1.03 (1.02, 1.05) | 0.0001 |
| hCG [median (IQR), IU/L] | - | 0.08 (0.00, 0.24) | 0.08 (0.00, 0.21) | 0.81 (0.44, 1.48) | 0.4905 |
| Prolactin [median (IQR), ng/mL] *^a^* | - | 9.10 (6.67, 14.04) | 7.87 (5.38, 12.08) | 1.04 (1.00, 1.08) | 0.0674 |
| DHEAS [median (IQR), μg/dL] *^a^* | - | 53.70 (33.10, 76.20) | 37.15 (15.20, 65.95) | 1.01 (1.00, 1.02) | 0.0046 |
| Testosterone [median (IQR), ng/dL] *^a^* | - | 8.76 (0.00, 20.41) | 0.00 (0.00, 9.44) | 1.06 (1.04, 1.09) | <0.0001 |
| Cortisol [median (IQR), μg/dL] | - | 6.63 (4.72, 10.65) | 6.74 (4.81, 10.40) | 0.99 (0.94, 1.04) | 0.6155 |
| ACTH [median (IQR), pg/mL] | - | 27.75 (20.00, 37.95) | 20.9 (15.5, 27.3) | 1.00 (1.00, 1.00) | 0.3917 |
| Total triiodothyronine [mean (SD), ng/dL] | - | 138.2 (22.9) | 141.8 (26.0) | 0.99 (0.99, 1.00) | 0.1969 |
| Free triiodothyronine [mean (SD), pg/mL] | - | 3.84 (0.55) | 3.87 (0.58) | 0.90 (0.60, 1.35) | 0.6044 |
| Total thyroxine [mean (SD), μg/dL] *^a^* | - | 8.79 (1.81) | 9.22 (1.83) | 0.88 (0.77, 0.99) | 0.0394 |
| Free thyroxine [mean (SD), ng/dL] *^a^* | - | 0.95 (0.18) | 0.99 (0.15) | 0.26 (0.06, 1.04) | 0.0575 |
| TSH [median (IQR), μIU/mL] | - | 2.17 (1.58, 2.99) | 1.83 (1.35, 2.91) | 1.08 (0.91, 1.28) | 0.3780 |
| Pelvic sonogram |  |  |  |  |  |
| Average ovarian volume (mL) *^a^* |  |  |  |  |  |
| <1mL | 9 (5.2) | - | - | 2.52 (1.74, 3.63) | <0.0001 |
| 1-<2mL | 82 (47.7) | - | - |  |  |
| $\geq$2mL | 81 (47.1) | - | - |  |  |
| Largest ovarian volume (mL) *^a^* |  |  |  |  |  |
| <1mL | 7 (4.1) | - | - | 2.78 (1.91, 4.06) | <0.0001 |
| 1-<2mL | 66 (38.4) | - | - |  |  |
| $\geq$2mL | 99 (57.6) | - | - |  |  |
| Smallest ovarian volume (mL) *^a^* |  |  |  |  |  |
| <1mL | 19 (11.1) | - | - | 2.72 (1.90, 3.89) | <0.0001 |
| 1-<2mL | 88 (51.2) | - | - |  |  |
| $\geq$2mL | 65 (37.8) | - | - |  |  |
| Uterine |  |  |  |  |  |
| Length (cm) *^a^* |  |  |  |  |  |
| <3cm | 142 (82.6) | - | - | 4.79 (1.93, 11.87) | 0.0003 |
| 3-<4cm | 30 (17.4) | - | - |  |  |
| $\geq$4cm | 0 (0.0) | - | - |  |  |
| Volume (mL) *^a^* |  |  |  |  |  |
| <3mL | 113 (65.7) | - | - | 3.27 (1.98, 5.39) | <0.0001 |
| 3-<4mL | 36 (20.9) | - | - |  |  |
| $\geq$4mL | 23 (13.4) | - | - |  |  |
| Endometrium visible (%) *^a^* |  |  |  | 3.83 (1.70, 8.61) | 0.0012 |
| No | 140 (81.4) | - | - |  |  |
| Yes | 32 (18.6) | - | - |  |  |

BMI: body mass index; LH: luteinizing hormone; FSH: follicle-stimulating hormone; hCG: human chorionic gonadotropin; DHEAS: dehydroepiandrosterone sulfate; ACTH: adrenocorticotropic hormone; TSH: thyroid - stimulating hormone. IQR: interquartile range.

*^a^* Predictor that was entered into the multivariable logistic regression model for the stepwise selection (*P* < 0.20).

Additional Table 2 Selected prediction model for central precocious puberty in the training sample (n=314)

| Predictors | β (SE) | Odds ratio (95% CI) | *P* value |
| --- | --- | --- | --- |
| Intercept | -6.42 (0.99) | - | <0.0001 |
| Age at onset of puberty | 0.45 (0.11) | 1.57 (1.25, 1.97) | <0.0001 |
| Basal LH | 1.63 (0.29) | 5.09 (2.88, 9.00) | <0.0001 |
| Largest ovarian volume | 0.66 (0.24) | 1.94 (1.20, 3.12) | 0.0066 |
| Uterine volume | 0.85 (0.32) | 2.33 (1.25, 4.34) | 0.0075 |

Additional Table 3 Potential prediction models for central precocious puberty in the training sample and validation sample

|  | Predictor(s) | AUC in the training sample (n=314) | AUC in the validation sample (n=313) |
| --- | --- | --- | --- |
| Model 1 | Age at onset of puberty | 0.70 | 0.68 |
| Model 2 | Basal LH | 0.82 | 0.84 |
| Model 3 | Largest ovarian volume+ Uterine volume | 0.70 | 0.73 |
| Model 4 | Age at onset of puberty+ Basal LH+ Largest ovarian volume+ Uterine volume | 0.86 | 0.86 |

AUC: area under the ROC curve.
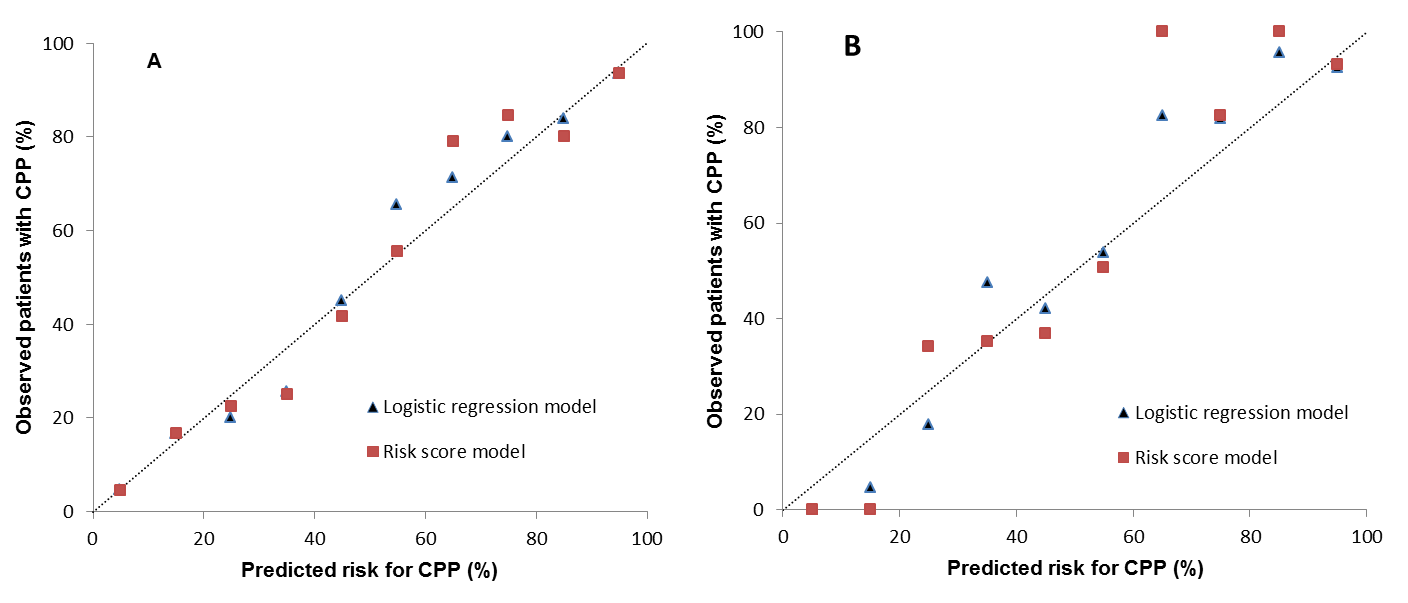


Additional Figure 1 Calibration plot for the selected logistic regression model and risk score model in the training and validation sample (A. Training sample B. Validation sample)


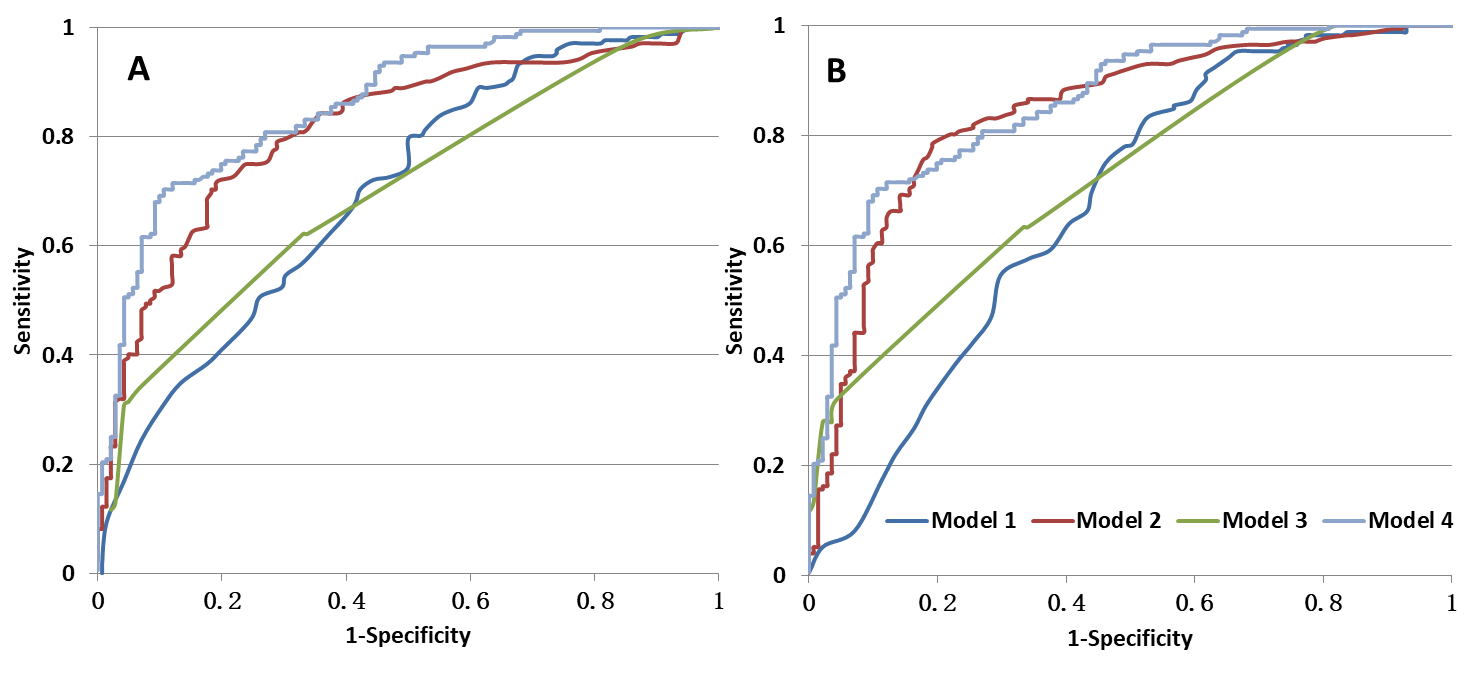


Additional Figure 2 Receiver operating characteristic curves of the prediction models (A. Training sample B. Validation sample)

Prediction models 1-4 were described in Additional Table 3.
