# Supplementary material for: Clinical risk score for central precocious puberty among girls with precocious pubertal development: a cross sectional study
Source: BMC Endocr Disord. 2021 Apr 20;21:75. doi: 10.1186/s12902-021-00740-7 (PMC8056580; doi:10.1186/s12902-021-00740-7)
Supplement: Supplementary file 2 — Additional file 2. Method: Risk score system. [file 12902_2021_740_MOESM2_ESM.docx]

**Method**

Risk score system

A risk score system based on the final multivariable model was derived using the method proposed by Sullivan et al.^1^ In the scoring system, 0 points were assigned to the base category (the reference value W_iREF_) for each risk category. We then calculated the distance of each category from the base category for each risk factor in terms of regression units [β_i_ (W_ij_-W_iREF_)]. We defined the constant for the point system, which correspond to one point in the risk score system, as the increase in risk of CPP associated with a 0.2 (IU/L) increase in basal LH: B=0.2*1.63=0.326. Point associated with each category of each predictor was calculated according to the formula: Points_ij_=βi (W_ij_-W_iREF_)/B. The points are rounded to the nearest integer (Appendix Table 2). The points total was calculated and multiplied by the constant (B=0.326). Based on the sum of total point, the estimate of the intercept, and the base values for each risk factor (onset age, basal LH, largest ovarian volume score, and uterine volume score) added back to the model, we estimated the risk of CPP according to the formula:

$\hat{p}=\frac{1}{1+exp(-\sum_{i=0}^{p} \beta_{i}X_{i})}$, $\sum_{i=0}^{p} \beta_{i}X_{i}$=-6.42+0.45*0.5+1.63*0.1+0.66*1+0.85*1+B*(Point total)].

**Reference**

1. Sullivan LM, Massaro JM, D'Agostino RB, Sr. Presentation of multivariate data for clinical use: The Framingham Study risk score functions. *Statistics in medicine.* 2004;23(10):1631-1660.
